# Mechanisms of the FLASH effect: current insights and advances

**DOI:** 10.3389/fcell.2025.1575678

**Published:** 2025-05-09

**Authors:** Giulia Rosini, Esther Ciarrocchi, Beatrice D’Orsi

**Affiliations:** ^1^ Institute of Neuroscience, Italian National Research Council, Pisa, Italy; ^2^ Department of Physics, University of Pisa, Pisa, Italy; ^3^ National Institute of Nuclear Physics, Section of Pisa, Pisa, Italy; ^4^ University of Pisa, Center for Instrument Sharing of the University of Pisa (CISUP), Pisa, Italy

**Keywords:** radiotherapy, ultra-high-dose rate irradiation, flash, cell death, cancer metabolism, cancer cells

## Abstract

Radiotherapy is a fundamental tool in cancer treatment, utilized in over 60% of cancer patients during their treatment course. While conventional radiotherapy is effective, it has limitations, including prolonged treatment durations, which extend patient discomfort, and toxicity to surrounding healthy tissues. FLASH radiotherapy (FLASH-RT), an innovative approach using ultra-high-dose-rate irradiation, has shown potential in selectively sparing normal tissues while maintaining unaltered tumor control. However, the precise mechanisms underlying this “FLASH effect” remain unclear. This mini-review explores key hypotheses, including oxygen depletion, radical-radical interactions, mitochondrial preservation, differential DNA damage repair, and immune modulation. Oxygen levels significantly affect tissue response to radiation by promoting radical recombination, preserving mitochondrial function, and differentially activating DNA repair pathways in normal versus tumor tissues. However, the extent to which oxygen depletion contributes to the FLASH effect remains debated. Additionally, FLASH-RT may modulate the immune response, reducing inflammation and preserving immune cell function. To further enhance its therapeutic potential, FLASH-RT is increasingly being combined with complementary strategies such as radioprotectors, immunomodulators, and nanotechnology platforms. These combinations aim to amplify tumor control while further reducing normal tissue toxicity, potentially overcoming current limitations. Despite promising preclinical evidence, the exact mechanisms and clinical applicability of FLASH-RT require further investigation. Addressing these gaps is crucial for optimizing FLASH-RT and translating its potential into improved therapeutic outcomes for cancer patients. Continued research is essential to harness the full benefits of the FLASH effect, offering a paradigm shift in radiation oncology.

## 1 Introduction

Radiotherapy is one of the most effective treatments for cancer, used in more than 60% of cancer patients during their oncological care to eliminate/reduce the size of the tumor (Giannini et al., 2024). Currently, conventional radiotherapy (CONV-RT) remains the standard in clinical practice but has limitations, including the risk of damage to surrounding healthy tissues (Bourhis et al., 2019). A recent innovation, FLASH radiotherapy (FLASH-RT), employs ultra-high-dose rate (UHDR) irradiation to selectively spare healthy tissue while maintaining its therapeutic effect on tumors. However, the precise radiobiological mechanism behind this protective “FLASH effect” remains unclear (Alaghband et al., 2023). To understand the FLASH effect, several hypotheses have been proposed, focusing on the differential responses of normal and tumor tissues to UHDR irradiation: *(i)* Oxygen depletion: FLASH-RT may rapidly deplete oxygen in normal tissues, creating transient hypoxia that reduces oxygen-dependent DNA damage; (ii) Radical-radical interaction: The rapid production of reactive oxygen species (ROS) during UHDR irradiation may lead to radical recombination, preventing oxidative damage to healthy tissues; (iii) Mitochondrial preservation: FLASH-RT appears to preserve mitochondrial integrity and ATP production in normal tissues, minimizing oxidative stress. Conversely, FLASH-RT may promote oxidative damage and apoptosis in tumor cells, potentially improving therapeutic efficacy; (iv) DNA damage and repair: The differential response of normal and tumor tissues may result from variations in DNA damage formation and repair. Normal cells rely on highly conserved repair mechanisms, while tumor cells often exhibit dysregulated repair pathways; and (v) Immune response: FLASH-RT may better preserve circulating immune cells and reduce inflammation in normal tissues compared to CONV-RT. In this mini-review, we summarize the current insights into the cellular mechanisms underlying the FLASH effect. Preclinical studies in animal models have demonstrated the FLASH effect, and early-phase clinical trials are now underway to evaluate its safety and efficacy in human patients. While FLASH-RT holds great promise for improving the balance between tumor control and normal tissue sparing in cancer treatment, continued research is necessary to fully elucidate its mechanisms, optimize its clinical application, and minimize potential side effects. Understanding these mechanisms will pave the way for safer and more effective radiotherapy strategies.

## 2 Mini review

### 2.1 Physical principles of FLASH radiotherapy: mechanisms and delivery

The FLASH effect is achieved using UHDR beams, typically exceeding 40 Gy/s as average dose rate, with irradiation durations shorter than 100 milliseconds (ms). These beams are generally produced by dedicated linear accelerators (Di Martino et al., 2023) or reversibly modifying pre-existing commercial clinical systems originally designed for CONV-RT (Felici et al., 2020), with laser-driven electron acceleration also being a viable option (Labate et al., 2020). The most frequently studied particle types include low-energy (10 MeV) and very high-energy (150 MeV) electrons (Almeida et al., 2024a), as well as protons (Nesteruk and Psoroulas, 2021). Research has also explored the use of photons and carbon ions (Montay‐Gruel et al., 2022) (Weber et al., 2022). The differences between FLASH and CONV irradiation become evident when comparing a typical 10 Gy dose used in *in vivo* experiments. A comparison of FLASH and CONV irradiation parameters is provided in Table 1. While CONV irradiation delivers the dose over several minutes, FLASH achieves the same dose within a few hundred ms, resulting in significant differences in instantaneous dose rate and dose per pulse.

**TABLE 1:** Representative irradiation scheme for delivering a total dose of 10 Gy in both CONV and FLASH regimes.

Parameter	Description	CONV value	FLASH value
D	Total Dose	10 Gy	10 Gy
T	Total Irradiation Time	5 min	100 ms
<Ḋ>	Average Dose Rate	2 Gy/min	100 Gy/s
DPP	Dose Per Pulse	0.66 mGy	2 Gy
IDR	Instantaneous, Intra-Pulse Dose Rate	150 Gy/s	0.5 MGy/s
n	Number of Pulses	15,000	5

### 2.2 Oxygen depletion

Among the leading hypotheses explaining the FLASH effect, oxygen depletion initially emerged as a central focus of research due to its potential role in selectively protecting normal tissues from radiation damage. Preliminary studies suggest correlation between hypoxia and the FLASH effect, indicating that oxygen depletion may contribute to normal tissue protection, though this remains debated. FLASH-RT delivers ultra-high doses in milliseconds, which has been hypothesized to trigger a rapid reduction in oxygen concentration and induced transient hypoxia in normal tissues (Wilson et al., 2020; Grilj et al., 2024a). This hypoxic shift could enhance radioresistance by limiting the formation of oxygen-dependent DNA lesions, thereby protecting normal cells from irreversible radiation-induced damage. Unlike CONV-RT, which delivers radiation over minutes and allows for cellular reoxygenation, FLASH-RT administers doses in milliseconds, depleting local oxygen before replenishment (Scarmelotto et al., 2024). Notably, a study conducted on prostate cancer cells under varying oxygen concentrations demonstrated that hypoxia uniquely influenced tumor cell responses to FLASH irradiation compared to CONV-RT (Adrian et al., 2020). Radiation chemistry analysis further supported the role of hypoxia in tumor cell response to FLASH-RT compared to CONV, demonstrating that FLASH-RT may rapidly consume oxygen through radical-driven reactions, potentially inducing transient hypoxia (Wardman, 2023). Additionally, physics studies on water radiolysis following irradiation provided further evidence, demonstrating a faster reduction in oxygen concentration under FLASH-RT compared to CONV-RT (Kusumoto et al., 2020; Sunnerberg et al., 2023; Thomas et al., 2024; Cao et al., 2021). However, it is necessary to specify that this was an indirect evidence.

The strongest experimental evidence supporting the oxygen depletion hypothesis stemmed from a series of *in vivo* studies conducted in healthy murine models, which employed a reverse approach by increasing the oxygen concentration in irradiated tissues (Montay-Gruel et al., 2019; Zhang et al., 2023). In these experiments, the anticipated radioprotective hypoxia was not achieved, nullifying the benefits of FLASH-RT in an overly oxygenated brain. However, recent reports using more sensitive measurements of oxygen tension *in silico*, *in vitro*, and *in vivo* challenge this hypothesis, indicating that FLASH-induced oxygen depletion may be insufficient to fully account for its observed biological benefits (Cao et al., 2021; Jansen et al., 2021; El Khatib et al., 2022; Van Slyke et al., 2022). These findings suggest that doses approaching 100 Gy are generally necessary to induce even a modest (3%) reduction in oxygen levels (Michaels, 1986; Weiss et al., 1974), which significantly exceeds the ∼10 Gy typically required to elicit the *in vivo* FLASH effect. Moreover, a dose of 100 Gy is lethal, further highlighting the disparity between oxygen depletion and the dose range associated with FLASH-RT (Limoli and Vozenin, 2023). Moreover, *in vitro* and *in vivo* studies measuring oxygen consumption during FLASH-RT have suggested that oxygen depletion to radiologically relevant levels of hypoxia is unlikely to occur in bulk tissue under FLASH irradiation casting doubt on whether FLASH-induced oxygen depletion is substantial enough to explain its protective effects (Cao et al., 2021). Similarly, a computational modelling study suggests that the dose levels typically used in FLASH experiments are insufficient to induce significant oxygen depletion, and thus unlikely to impact radiosensitivity unless the tissue is already in a state of extreme hypoxia (Boscolo et al., 2021).

These conflicting findings raise critical questions about whether oxygen depletion alone can fully explain the FLASH effect (Pogue et al., 2024; Swartz et al., 2022). Instead, alternative mechanisms, such as differential ROS modulation, immune response, or metabolic adaptations, may play a more significant role.

### 2.3 Radical-radical interaction

Ionizing radiation (IR) in CONV-RT induces DNA damage through both direct and indirect mechanisms. The direct effect involves the formation of organic radicals on DNA bases and other biomolecules, such as proteins and lipids. The indirect effect arises from water radiolysis, generating ROS that account for approximately two-thirds of biomolecular damage, including DNA strand breaks (Jansen et al., 2022). Key ROS include hydroxyl radicals (·OH), superoxide (O2·−), hydrogen peroxide (H_2_O_2_), and organic peroxides (ROO· or ROOH), which can interact with labile iron (Fe^2+^) to exacerbate oxidative damage. Oxygen plays a critical role in stabilizing radiation-induced radicals, leading to permanent biomolecular lesions (Ibáñez et al., 2024).

For a given dose, the number of ionization events remains constant between FLASH-RT and CONV-RT. However, the UHDR irradiation generates a substantially higher concentration of free radicals within a much shorter time frame compared to CONV-RT, potentially altering their interaction. One hypothesis suggests that UHDR irradiation rapidly depletes oxygen in tissues, thereby reducing the propagation of radiation-induced damage mediated by ROS (Eric, 2018). Unlike tumor cells, which often exhibit high endogenous ROS production, normal cells generally have greater antioxidant capacity to neutralize ROS (Nakamura and Takada, 2021). It is hypothesized that the antioxidant reserves in normal tissues help eliminate ROS generated by FLASH-RT before they trigger Fenton reactions and lipid peroxidation, thereby reducing toxicity (Geirnaert et al., 2025). As a result, normal tissues may experience lower accumulation of ROO· and ROOH compared to tumors, which frequently have impaired antioxidant defenses. FLASH-RT may cause a temporary surge of organic radicals (R•) and organic peroxyl radicals (ROO•), which are more likely to recombine and form stable, non-radical compounds. Increased radical recombination has been suggested as a potential mechanism that shortens redox chain reactions. This process may limit oxidative damage to healthy tissues, by reducing protein and lipid peroxidation, as well as DNA damage (Geirnaert et al., 2025). Spitz et al. proposed that the FLASH effect arises from differences in antioxidant capacity between mouse tumor and normal tissues, with UHDR irradiation amplifying these differences (Spitz et al., 2019). Specifically, normal tissues, which have lower pool of labile iron (limiting Fenton-type reactions) and faster radical detoxification, would experience lower concentrations of organic hydroperoxides compared to tumor tissues when exposed to UHDR (Spitz et al., 2019). Other hypotheses closely align with this latest report indicating that the tissue-sparing effects of FLASH-RT are linked to oxygen depletion and/or lipid peroxidation, ultimately limiting the Fenton reaction and ferroptosis, an iron-dependent form of cell death (Vilaplana-Lopera et al., 2022; Scarmelotto et al., 2024). In contrast, under CONV irradiation, this differential response diminishes, as the antioxidant systems in both normal and tumor tissues effectively neutralize the radicals generated during each irradiation pulse. Moreover, a physicochemical model suggests that UHDR irradiation induces a transient surge in peroxyl radicals, promoting their recombination and thereby reducing oxidative stress in the irradiated volume (Labarbe et al., 2020). This theory further posits that the differential effects of UHDR irradiation on normal and tumor tissues stem from variations in antioxidant capacity. In normal tissues, the physiological antioxidant pool becomes rapidly saturated due to radiation-induced ROS production, limiting its ability to fully counteract cytotoxic effects (Hu et al., 2023). However, during UHDR irradiation, rapid radical recombination can reduce overall ROS levels, mitigating damage. This protective effect is less pronounced in tumor tissues, where higher antioxidant reserves enable more effective neutralization of oxidative stress, leading to comparable cytotoxic outcomes under both CONV and UHDR irradiation. Further experimental studies by the Froidevaux research group (Froidevaux et al., 2023; Grilj et al., 2024b) used linoleic acid micelles and phosphatidylcholine (PC) liposomes as models of the cell membrane to investigate lipid peroxidation yields following FLASH and CONV irradiation. These studies aimed to determine whether lipid peroxidation end products differed significantly between the two irradiation modalities. The results revealed that lipid micelles and PC liposomes exhibited a linear, dose-dependent increase in lipid peroxidation with CONV irradiation, whereas FLASH irradiation did not induce measurable lipid peroxidation. These findings suggest that lipid oxidation may be a critical determinant of the FLASH effect, providing a potential explanation for its selective protection of normal tissues while maintaining tumor cytotoxicity.

### 2.4 Mitochondrial preservation

Mitochondria are essential for energy production, apoptosis regulation, and oxidative stress control, functions that are often dysregulated in cancer cells to promote uncontrolled proliferation. A hallmark of cancer is the Warburg effect, a metabolic shift from oxidative phosphorylation (OXPHOS) to aerobic glycolysis, even in the presence of oxygen (Glasauer and Chandel, 2014). This metabolic reprogramming enables cancer cells to generate biosynthetic precursors, such as nucleotides and amino acids, via glycolysis, regardless of oxygen availability. Although glycolysis generates less ATP per glucose molecule than OXPHOS, it provides a selective advantage in the hypoxic tumor microenvironment by allowing rapid ATP production and metabolic flexibility (Kam and Banati, 2013). OXPHOS generates ATP and ROS, such as superoxide (O_2_•−), which can react with nitric oxide and participate in Fenton chemistry, forming toxic species like peroxynitrite (ONOO−) and ·OH (Wardman, 2023). Although mitochondria possess antioxidant systems to regulate ROS levels, excessive radiation-induced ROS can overwhelm these defenses, leading to mitochondrial damage and triggering a self-perpetuating cycle of increased ROS production in cells. This damage leads to mitochondrial degradation via mitophagy and outer membrane permeabilization, mediated by pro-apoptotic proteins BAX and BAK, promoting cytochrome c release (Marchi et al., 2023). Beyond its role in the electron transport chain, cytochrome c activates apoptotic caspases. The release of mitochondrial ROS and DNA can activate inflammatory pathways, further contributing to cellular dysfunction (Averbeck and Rodriguez-Lafrasse, 2021; D’Orsi et al., 2017). Mitochondrial electron transport may be highly sensitive to ionizing radiation, particularly at complex I and III, where ROS production would be amplified. CONV-RT and FLASH-RT might exert distinct effects on mitochondrial metabolism and cell death in normal and tumor tissues. In normal tissues, CONV-RT may increase ROS production, including ∙OH, which form organic peroxides ∙ROOs to ROOHs, potentially disrupting OXPHOS, reducing ATP synthesis, and causing elevated mitochondrial ROS (mtROS) levels that could lead to damage, cytochrome c release, and apoptosis or necrosis (Geirnaert et al., 2025). CONV-RT also promotes mitochondrial fission through *Drp1* activation in cells (Guo et al., 2022). Despite these effects, normal tissues would maintain a predominantly OXPHOS-dependent metabolic profile. Supporting this hypothesis, Ren et al. demonstrated the protective effect of FLASH-RT on esophageal tissue compared to CONV-RT. Histopathological analysis revealed significantly less tissue damage in FLASH-irradiated mice than in those treated with CONV-RT. Furthermore, label-free quantitative proteomic analysis indicated that this protective effect was linked to reduced protein damage associated with mitochondrial functions and a diminished acute inflammatory response. These findings suggest that the tissue-sparing effect of FLASH-RT may occur through alleviated mitochondrial damage and reduced acute inflammation (Ren et al., 2024). Moreover, in normal human lung fibroblasts, it has been demonstrated that FLASH-RT preserves Drp1 phosphorylation, thereby preventing excessive mitochondrial fission and necrosis (Guo et al., 2022). This protective effect may allow normal tissues to retain their predominantly OXPHOS-dependent metabolic state (Alhaddad et al., 2024).

In tumor tissues, FLASH-RT, similarly to CONV-RT may increase ROS levels, disrupting OXPHOS and ATP production, promoting mtROS accumulation, and causing mitochondrial damage (Guo et al., 2022). As with CONV-RT, this could trigger cytochrome c release, leading to a stronger apoptotic response rather than necrosis. FLASH-RT also promotes mitochondrial fission through *Drp1* activation (Guo et al., 2022). Under these conditions, tumor metabolism may shift from OXPHOS to glycolysis, aiding adaptation to hypoxia and radiation-induced stress (Nakamura and Takada, 2021). In both normal and tumor cells, these changes may trigger cytochrome c release, ultimately inducing apoptosis and necrosis (Han et al., 2021). However, tumor cells often respond to hypoxia and ionizing radiation by shifting their metabolism from OXPHOS to glycolysis, enhancing their survival under these conditions (Alhaddad et al., 2024). In contrast, FLASH-RT might generate lower ROS levels in normal tissues, preserving mitochondrial integrity, maintaining OXPHOS and ATP production, and limiting mtROS accumulation. Consequently, cytochrome c release may be reduced, favoring apoptosis over necrosis, which could help minimize inflammatory damage to surrounding tissues.

These findings suggest that FLASH-RT may offer a therapeutic advantage over CONV-RT by preserving mitochondrial function and metabolism integrity in healthy tissues while promoting tumor control through oxidative stress and selective apoptosis induction (Geirnaert et al., 2025). Although these findings are preliminary and require further validation across various normal tissues and tumor types, they indicate that mitochondrial metabolic alterations might play a key role in driving the FLASH effect.

### 2.5 DNA damage and repair

Unrepaired DNA damage, particularly double-strand breaks (DSBs), is a critical factor in the cellular response to IR exposure (Scott and Pandita, 2006). The differential response of tumor and normal tissues to FLASH-RT may arise from differences in radiation-induced damage formation and repair mechanisms (Labarbe et al., 2020). Unlike tumor cells, which often have dysregulated DNA repair pathways, normal cells possess highly conserved mechanisms, including Non-Homologous End Joining (NHEJ) and Homologous Recombination (HR), which predominate in late S and G2 phases (Toulany, 2019). Alterations in DNA repair pathways or cell cycle checkpoints can modulate the response to IR. Poly (ADP-ribose) polymerases (PARPs), chromatin-associated enzymes, play a crucial role in chromatin regulation, replication, transcription, DNA repair, and the innate immune response (Malanga and Althaus, 2025). The catalytic activity of PARP-1,-2, and -3 isoforms is stimulated by single-strand breaks, promoting their repair (Malanga and Althaus, 2005). DSBs can activate the cyclic GMP-AMP synthase (cGAS) sensor and its downstream effector, STING, a key innate immune pathway that detects cytosolic DNA as a danger signal. This triggers the production of type I interferons (IFNs) and inflammatory cytokines, leading to cellular senescence, autophagy, cell death, or tissue damage (Lv et al., 2024). Activation of the cGAS–STING pathway has been linked to increased tumor immunogenicity and enhanced dendritic cell activity with varying influence depending on tumor type, stage, and immune microenvironment (Zhou et al., 2023).

Dysregulation of chemokine and inflammatory cytokine secretion (e.g., TGF-β, IL-6) following CONV-RT has been associated with radiation-induced pulmonary fibrosis. In contrast, differential activation of the cGAS–STING pathway between normal and tumor cells may underlie the FLASH effect, potentially inhibiting tumor growth while protecting normal tissues from severe damage (Zhou, 2020). However, no study has conclusively demonstrated that differences in DNA damage alone are sufficient to explain the FLASH effect. While some studies report fewer DNA damage foci after UHDR irradiation at doses >20 Gy (Cooper et al., 2022), particularly in the early post-exposure phase, others find no significant differences in DSB induction between FLASH-RT and CONV-RT (Nair et al., 2019). Of note, γ-H2AX foci formation in irradiated normal fibroblasts and tumor cells resulted independent of the irradiation mode. In contrast, 53BP1 foci exhibited significant differences exclusively in normal cells, suggesting distinct DNA repair mechanisms involving 53BP1 (Buonanno et al., 2019; Fouillade et al., 2020). DNA damage foci were analyzed in lung cells isolated from irradiated mice at 1 week and 3 months post-exposure, with age-matched non-irradiated lungs serving as controls. At the 1-week time point, both CONV-RT and FLASH-RT exposed cells exhibited elevated DNA damage compared to controls, as evidenced by increased numbers of 53BP1-positive cells and higher foci counts per cell. Notably, CONV-RT samples demonstrated significantly greater damage levels than FLASH-RT across both parameters. By 3 months post-irradiation, a clear divergence emerged between the treatment groups. While FLASH-RT samples showed a reduction in foci per cell, CONV-RT samples displayed the opposite trend, with increasing foci numbers over time. Furthermore, the proportion of damage-positive cells remained stable in FLASH-irradiated samples but rose significantly in CONV-RT treated animals. This persistent accumulation of DNA damage in the CONV-RT group, occurring well beyond the initial radiation exposure, may suggest ongoing genomic instability in the absence of additional exogenous injury (Fouillade et al., 2020). Further studies are needed to elucidate the mechanisms underlying this differential response (Osipov et al., 2023). Investigations into the unique molecular and cellular processes activated by FLASH-RT may help optimize therapeutic outcomes while minimizing damage to healthy tissues.

### 2.6 Immune response

Radiation therapy can induce both pro-inflammatory and immunosuppressive responses, highlighting the complex interplay radiation effects and the immune system. FLASH-RT has been shown to reduce inflammation in normal tissues while enhancing antitumor immunity in murine models, possibly preserving circulating immune cells and modulating the tumor microenvironment (TME) (Zhu et al., 2023). The increased infiltration of T lymphocytes in tumors following FLASH-RT, compared to CONV-RT, supports the hypothesis that FLASH-RT directly enhances the anti-tumor immune response rather than merely sparing healthy tissues (Kim et al., 2017; Rama et al., 2019). In murine models of lung adenocarcinoma, FLASH-RT and CONV-RT showed similar efficacy in delaying tumor growth, regardless of immune status. However, the immune response, particularly T-cell activation and cytokine modulation, is thought to contribute to a portion of FLASH-RT’s antitumor efficacy (Almeida et al., 2024b). The computational model developed by Jin et al. showed that FLASH-RT significantly reduces the loss of circulating immune cells, preserving immune function and tissue repair capacity. Specifically, a single dose ≥30 Gy delivered via FLASH-RT reduced the loss of circulating immune cells to 5%–10%, whereas CONV-RT resulted in a depletion of 90%–100% (Jin et al., 2020). In a healthy murine model, it has been demonstrated that FLASH-RT reduces neuroinflammation, as evidenced by lower pro-inflammatory cytokine levels and reduced activation of CD68-positive microglia compared to CONV-RT (Simmons et al., 2019). The TME, including immune cell composition and cytokine signaling, plays a critical role in determining radiation efficacy. Recent studies suggest that tumor vascular collapse and immune cell infiltration are key factors influencing radiotherapy outcomes. For instance, Kim et al. demonstrated that FLASH-RT, unlike CONV-RT, prevents tumor vascular collapse, indicating a differential impact on tumor vascularization (Kim et al., 2021). Tumor vascular collapse may impair tumor perfusion and oxygenation, ultimately affecting treatment response (Zhang et al., 2022). This effect may contribute to the enhanced antitumor efficacy of FLASH-RT, particularly when compared to CONV-RT, which can exacerbate vascular damage and tumor hypoxia. Additionally, FLASH-RT has been shown to modulate the immune microenvironment within tumors. While it induces immune responses (e.g., lymphocytic infiltration) similar to CONV-RT, the dynamics of immune activation and suppression may differ (Ma et al., 2024). The combination of improved vascular preservation and modulation of the immune response may contribute to the superior efficacy of FLASH-RT in certain tumors. However, the full extent of its effects on tumor immune infiltration remains an active area of research.

Particular attention has been given to the role of the cytokine TGF-β, a key regulator of the radiation-induced tumor responses, which has been found to exhibit distinct activation patterns under FLASH-RT and CONV-RT (Favaudon et al., 2014). Experiments on normal human lung fibroblasts suggest that FLASH-RT at high dose rates reduces TGF-β pathway activation compared to lower dose rates. Radiation exposure induces immune factors, including interleukins, interferons, and immune checkpoint ligands, which may mediate the bystander effects (localized immune response to irradiated cells) and contribute to abscopal effects (systemic immune activation against distant tumors). It is hypothesized that abscopal effects are mediated by T lymphocytes, with macrophages playing a crucial role in inflammatory responses and radiation-induced immunogenic cell death (Golden and Apetoh, 2015). Specifically, immunogenic cell death may be driven by cGAS-STING pathway activation and cytosolic DNA release, triggering an innate immune response (Shen et al., 2021). While preliminary findings suggest FLASH-RT significantly impacts the TME and immune response, further studies are needed to elucidate the underlying mechanisms and whether these effect translate into clinically meaningful benefits.

### 2.7 FLASH-RT in combination with other therapies

Despite its therapeutic advantages, FLASH-RT still faces challenges, such as tumor cell resistance and recurrence risk. To maximize therapeutic efficacy, FLASH-RT is increasingly being combined with complementary approaches. Current research focuses on three promising strategies: radioprotectors, including AsiDNA™ to enhance tumor radiosensitivity while sparing normal tissues; immunostimulatory agents (e.g., TLR agonist-loaded hydrogels) to boost antitumor immunity; and nanotechnology platforms, such as TAFL for targeted drug delivery. The first integrated approach is the employment of AsiDNA™, an oligonucleotide that mimics DSBs, disrupts tumor cell repair mechanisms, increasing their radiosensitivity. Notably, it also protects healthy tissues by reducing radiation-induced toxicity (Sesink et al., 2024). A study by Sesink et al. investigated AsiDNA™'s radioprotective effects when combined with both CONV-RT and FLASH-RT. Using murine models of radiation-induced pulmonary fibrosis, they found that AsiDNA™ triggers G1/S cell cycle arrest via the DNA-PK/p53/p21 pathway in epithelial cells and fibroblasts, improving cell survival post-irradiation. The FLASH effect was clearly confirmed, with results showing reduced early and late toxicity compared to CONV-RT. Interestingly, the combination of AsiDNA™ with CONV-RT was associated with greater late toxicity than FLASH-RT alone, despite showing comparable efficacy in mitigating early toxicity. This outcome might stem from potential limitations of AsiDNA™ in modulating the complex and not yet fully characterized mechanisms underlying late-onset tissue responses. Notably, the combination of AsiDNA™ with FLASH-RT failed to demonstrate any additive protective effect against either early or late toxicity compared to FLASH-RT alone, highlighting the need for further studies to elucidate optimal combination strategies (Sesink et al., 2024).

Moreover, immunotherapy, a standard treatment for cancers like melanoma, can be enhanced by combining it with RT. One innovative approach involves a radiopaque, radiation-responsive hydrogel (AuNP-IMQ-gel) loaded with the TLR7 agonist imiquimod (IMQ) and combined with FLASH-RT (Dong et al., 2023). In preclinical studies, FLASH-RT triggered rapid hydrogel degradation, releasing IMQ and inducing a potent immunostimulatory response. This combination significantly suppressed tumor growth and prolonged survival in murine melanoma models, both in melanoma cells and in xenograft. The released IMQ boosted pro-inflammatory cytokines (TNF-α, IL-6) and activated CD8^+^ T cells, demonstrating a strong synergistic effect (Dong et al., 2023).

Further innovations include emerging nanotechnology strategies to combat resistance and recurrence. To address tumor recurrence, particularly due to surviving cancer stem cells (CSCs), researchers are developing advanced nanoplatforms that synergize with FLASH-RT. Two innovative approaches show exceptional promise: the TAFL, a biomimetic nanoplatform targeting CSCs, and biomimetic nanoparticles and photothermal synergy. TAFL is a hybrid nanosystem combining tumor-derived exosomes with liposomes, loaded with the photothermal agent TPE-BBT and aspirin. This platform leverages photothermal-triggered drug release to target CSCs and impair DNA repair mechanisms, remodel the immunosuppressive tumor microenvironment and reduce metastasis and recurrence in aggressive cancers (Suo et al., 2024). In triple-negative breast cancer models, FLASH-RT + TAFL drastically reduced tumor recurrence and lung metastases compared to RT alone. The combination also decreased CD133+ CSCs, which are linked to treatment resistance and poor prognosis (Suo et al., 2024).

Beyond TAFL, emerging nanotechnology-driven strategies are showing promise for combinatorial FLASH-RT approaches. One innovative example involves platelet cell membrane-camouflaged hollow TaOx nanospheres encapsulating aggregation-induced emission luminogens (AIEgen), designated as TPT nanoparticles. These biomimetic constructs mediate photothermal therapy (PTT) while synergizing with FLASH-RT to enhance ROS production and induce ferroptosis in CSCs. Their platelet membrane coating enables deep tumor penetration, selectively eliminating residual CSCs and reducing recurrence risk (Lyu et al., 2023). Another promising formulation, termed CQu, has demonstrated potential to sensitize colorectal cancer models to FLASH-RT. As recently demonstrated by Lyu et al., CQu operates through a dual-action mechanism combining Photodynamic therapy (PDT)-mediated ROS generation and Ca^2+^ overload induction. This combinatorial approach may create a potent synergistic effect where oxidative stress and calcium dyshomeostasis markedly enhance tumor cell vulnerability to FLASH irradiation. Importantly, the treatment preserves FLASH’s characteristic tissue-sparing properties while simultaneously addressing the critical challenge of tumor recurrence prevention (Lyu et al., 2025).

Moreover, Shen et al. developed a light-activated hydrogel loaded with TPE-BBT and the glutaminase inhibitor CB-839. When exposed to 660 nm near-infrared light, the system generates mild hyperthermia, enhancing FLASH-RT’s radiosensitizing effects. In colorectal cancer models, this combination reduced recurrence while maintaining systemic safety (Shen et al., 2024).

In summary, the strategic combination of FLASH-RT with emerging modalities, including radioprotective agents, immunotherapies, and nanotechnology, may represent a paradigm shift in cancer treatment. These synergistic approaches could not only potentiate FLASH-RT’s antitumor efficacy but also augment its hallmark tissue-sparing effects, potentially redefining therapeutic outcomes for radiation oncology. Notably, further exploration of FLASH-RT combinations with other therapies, particularly those capable of amplifying its normal-tissue protection while preserving (or even enhancing) tumor control, could unlock novel mechanisms to widen the therapeutic window. Such dual-benefit strategies would be of high clinical relevance, offering both improved safety and efficacy.

### 2.8 Conclusion

**FIGURE 1:**
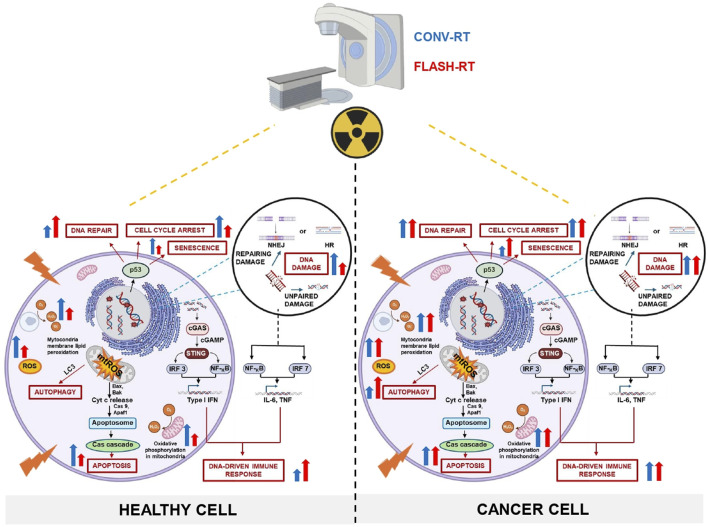
Comparative mechanisms of CONV-RT and FLASH-RT in cancer versus healthy cells: schematic integration of experimental evidence and current hypotheses.The figure illustrates the impact of CONV and FLASH irradiation modalities on DNA repair and damage, mitochondrial injury, cell cycle arrest, senescence, autophagy, apoptosis, and immune responses in both healthy and cancer cells. Moreover, the scheme also highlights the potential for FLASH-RT to selectively target cancer cells while sparing healthy tissues, thereby reducing collateral damage and improving therapeutic outcomes. CONV-RT delivers radiation at a standard dose rate, typically in the range of a few Gy per minute, while FLASH-RT administers radiation at an UHDR, often exceeding 40 Gy per second. However, oxygen depletion alone may not fully account for the observed biological benefits. Additional mechanisms, such as ROS modulation, immune responses, and metabolic alterations, likely may play significant roles. The figure emphasizes the critical role of ROS in mediating the differential effects of FLASH-RT. Tumor cells, which typically exhibit elevated levels of endogenous ROS, experience oxidative damage due to radical accumulation. In contrast, normal tissues contain robust antioxidant reserves that rapidly neutralize ROS, reducing the formation of harmful peroxidized compounds, such as peroxyl radicals and organic peroxides. This protects normal cells from oxidative damage to proteins, lipids, and DNA. Following UHDR irradiation, normal cells exhibit lower ROS levels compared to CONV-RT, which helps preserve mitochondrial integrity, oxidative metabolism, and ATP production. As a result, cellular energy is maintained, and the release of cytochrome c, a key promoter of apoptotic cell death, is reduced, favoring the survival of healthy cells. In contrast, CONV-RT increases mtROS production, leading to mitochondrial damage, fission, and a heightened risk of apoptosis or necrosis. In tumors, UHDR irradiation induces mtROS accumulation, causing mitochondrial damage and enhancing the apoptotic response, ultimately aiding tumor control. Unrepaired DNA damage, particularly DSBs, may plays a crucial role in the cellular response to IR. The differential DNA repair mechanisms between tumor and normal cells, including the activation of the cGAS-STING pathway, may contribute to the FLASH effect. This pathway promotes immunogenic cell death and stimulates innate immune responses, potentially enhancing tumor immunogenicity while safeguarding normal tissues. In summary, both CONV-RT and FLASH-RT effectively damage cancer cells, but FLASH-RT may offer a more rapid and potent effect due to its UHDR. Importantly, FLASH-RT demonstrates a potential protective effect on healthy cells, minimizing radiation-induced damage compared to CONV-RT, which can cause significant harm to surrounding normal tissues.

FLASH-RT has emerged as a promising alternative to CONV-RT, offering potential advantages in reducing normal tissues toxicity while maintaining or even potentially enhancing tumor control. However, the underlying mechanisms remain incompletely understood. Oxygen depletion, radical recombination, mitochondrial preservation, DNA repair and immune response modulation, have all been proposed as contributing factors (summarized in Figure 1), but no single mechanism fully explains the FLASH effect. This further highlights the complex interplay between physical, biological, and immunological factors that might behind the FLASH effect. Importantly, combining FLASH-RT with adjuvant therapies, such as radioprotectors, immunotherapy or nanotechnology, could synergize with these mechanisms to further widen the therapeutic window. FLASH-RT’s ability to reduce inflammation, preserve immune function, and minimize damage to healthy tissues contrasts sharply with CONV-RT, which often induces significant toxicity. However, despite promising preclinical findings, critical questions remain regarding the precise mechanisms driving the FLASH effect and its clinical applicability. Continued research is essential to fully elucidate these mechanisms, optimize FLASH-RT delivery, and translate its benefits into safe and effective clinical applications. By addressing these challenges, FLASH-RT has the potential to significantly improve therapeutic outcomes for cancer patients, offering a paradigm shift in radiation oncology.
